# Requirement of artificial intelligence technology awareness for thoracic surgeons

**DOI:** 10.1186/s43057-021-00053-4

**Published:** 2021-07-03

**Authors:** Anshuman Darbari, Krishan Kumar, Shubhankar Darbari, Prashant L. Patil

**Affiliations:** 1 CTVS Department, AIIMS, Rishikesh, 249203 India; 2 CSE Department, National Institute of Technology, Srinagar, Uttarakhand 246174 India; 3 CSE, Technical University, Delft, Netherlands

**Keywords:** Artificial intelligence, Deep learning, Machine learning, Thoracic surgery, Robotic surgery, Thoracic cancers

## Abstract

**Background:**

We have recently witnessed incredible interest in computer-based, internet web-dependent mechanisms and artificial intelligence (AI)-dependent technique emergence in our day-to-day lives. In the recent era of COVID-19 pandemic, this nonhuman, machine-based technology has gained a lot of momentum.

**Main body of the abstract:**

The supercomputers and robotics with AI technology have shown the potential to equal or even surpass human experts’ accuracy in some tasks in the future. Artificial intelligence (AI) is prompting massive data interweaving with elements from many digital sources such as medical imaging sorting, electronic health records, and transforming healthcare delivery. But in thoracic surgical and our counterpart pulmonary medical field, AI’s main applications are still for interpretation of thoracic imaging, lung histopathological slide evaluation, physiological data interpretation, and biosignal testing only. The query arises whether AI-enabled technology-based or autonomous robots could ever do or provide better thoracic surgical procedures than current surgeons but it seems like an impossibility now.

**Short conclusion:**

This review article aims to provide information pertinent to the use of AI to thoracic surgical specialists. In this review article, we described AI and related terminologies, current utilisation, challenges, potential, and current need for awareness of this technology.

## Background

Scientific advances from multiple disciplines have already played a significant role in every branch of medicine, including thoracic surgery. The researches and techniques derived from more fields are usually required to solve problems and advancements. It is the rapid development of physics, chemistry, and biochemistry supported by mathematics, which has made various medical advances possible: Whether it is the development of optical physics for microscope and telescope, the discovery and use of radiation physics, or the advancements in the field of clinical laboratory and pathology investigations. Therefore, every thoracic surgeon must have a basic understanding of the fundamental science with recent advances to obtain a multi-dimensional progressive view of the field. New scientific advancements will help thoracic surgeons to expand an existing skillset and perform new innovative procedures.

While it is a known fact that each generation of physicians significantly exceeds the previous generation's accomplishments based on previous discoveries. Nonetheless, modern science is accelerating quicker than ever due to the advent of computer science, especially artificial intelligence (AI) development, and it seems we are on the edge of another revolution. Future surgeons will need to know and grasp a broader range of the modern technology advancements quicker than ever before [1].

The Oxford Dictionary defines “AI” as the development of computer machine systems to accomplish tasks requiring human brain intelligence such as visual perception, speech recognition, decision-making, and language translation [2]. AI-based technologies mainly focus on the machine which allows the computers to learn from data and interpret, based on a predefined algorithm and reproduce near-human brain interpretations. The networks in AI systems are designed to mimic the same way as human brain processes information and derive solutions. Multiple of these neural networks with base algorithms work together to get a final decision. AI-based technologies are already prevalent in many areas of our daily life. Google Maps uses AI to pick up traffic patterns and create efficient routes dynamically, and smartphones use AI to recognise facial and voice-based commands for routine daily tasks such as internet data searches and speech recognitions. But in the healthcare industry, its extensive use is still limited and started recently. Thoracic surgeons should be aware of this technological advancement and recent development that could augment their clinical practice.

The MEDLINE (Ovid) and PubMed databases were searched for articles and papers (Books and Documents, Clinical Trial, Meta-Analysis, Randomized Controlled Trial, Review, Systematic Review) for the last 5 years that included keywords from both of two categories: thoracic surgery and artificial intelligence (keywords: artificial intelligence, machine learning, neural network, computer-assisted surgery) with search filters applied as above. A total of 41 publications were found, and further from this list, articles related to cardiac surgery, echocardiography, mitral valve repair, and trans-catheter cardiac treatment modalities were excluded. The exact best match was found for 3 articles only. We present the following article in accordance with the journal’s review reporting checklist.

## Main text

### Basics of technology/terminology

The field of AI was started at Dartmouth College (Hanover, NH, USA) in 1956, where a group of computer scientists assembled to discuss mathematical theorems, language processing, game theory and how computers learn from analysing training examples [3]. At first, a rules-based system governed AI. But by the 1980s, medical uses were noted and experimented. AI is a mixture of computer science and robotics, but it focuses more on empirically processing machine behaviour with output. Essentially, the human programmer defined what the computer has to do. Now, AI-based technologies uses adaptive analytical power with autonomous learning and complex algorithms. Algorithms that could represent medical knowledge are coded into the system as data and provide direction for different clinical scenarios. Pattern recognition also allows AI to separate anatomical structures for visualisation and analysis. So, now AI-based systems can recognise patterns, anticipate future events, make sound decisions, learn and unlearn from mistakes, assist clinical decision-making, and discover relevant information from data.

Machine learning (ML) is a subfield of AI in which statistical models are used to learn patterns from data to accomplish a specific task. With ML, modern systems are considered more intelligent because they use algorithms that enable the machine (or Computer) to learn functions from one particular and potentially ever-changing dataset. ML algorithms can be divided into three types: supervised learning, unsupervised learning, and reinforcement learning. ML techniques range from simple linear models such as logistic regression and naïve Bayes classifiers to complex neural network models with many parameters. In this, the computer programme can somewhat learn by itself, and software can automatically adapt its behaviour to match that specific task’s requirements [4].

**Fig. 1:**
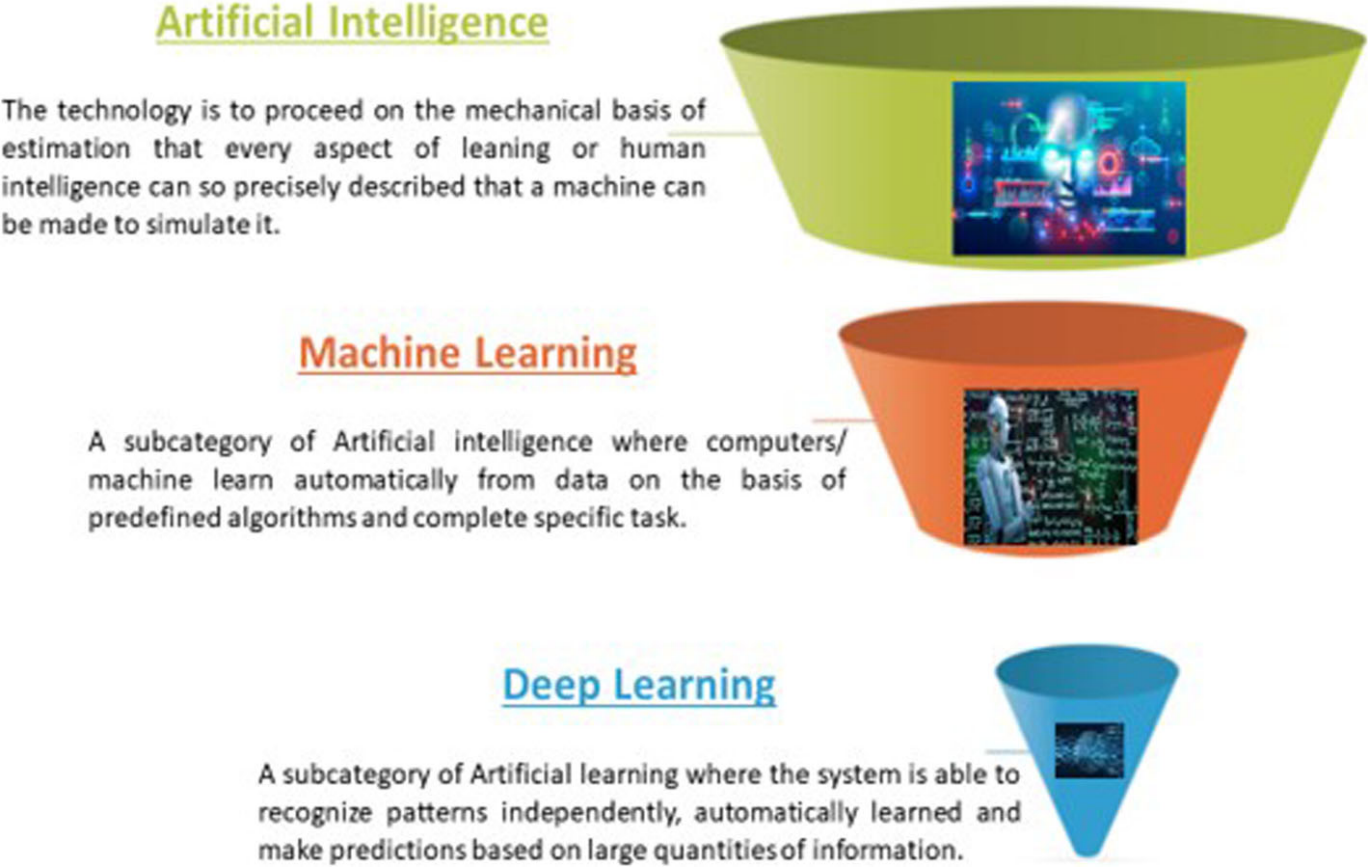
Various fields of artificial intelligence and definitions

Deep learning (DL) is a more inner subfield of the AI branch in which features needed for particular task completion are automatically learned from the raw data. But, DL requires costly and time-consuming human effort to accumulate raw data, provide statistical analysis and domain expertise, and build prior experimental models with specific feature sets and potentially complex algorithms. DL often overtakes other ML modalities when these massive datasets are manipulated. The development of DL is closely related to neural networks. It usually relies on principles similar to the human brain's working, composed of input and output layers of data representations called neurons. There are also various buried layers (neurons that cannot be defined in any specific either input or output layers). All layers are arranged sequentially so that a representation of one layer is fed into the following layer, the depth of the network being linked to the number of hidden and output layers. In more complex and deep networks, more powerful models to learn complex nonlinear mappings can be developed. These deep learning networks are trained by the method of ‘iteration’, which is a process of running and re-running networks with continuous optimising neuronal parameters to improve performance and minimise errors. Massive datasets drive this technological improvement with a pursuit for better algorithms and faster computational hardware. Figure 1 is the compiled representation of the various definitions in the AI field.

The explosion of interest in medical applications of AI and mainly DL during recent years may also be attributed to the convergence of especially two key factors:

1. Big data analysis and faster computation
2. Artificial neural networks (ANNs) or deep neural networks (DNNs): these networks are loosely modelled on the human brain mechanism, and consist of multiple layers of ‘neurons’ that successively process input data until the output layer is reached. DNNs are a recently developed variant of ANNs that have a large number of intermediate buried layers (often more significant than 10) and process input data hierarchically, with the first few layers responding to simple low-level features (such as straight lines) and successive layers responding to more abstract high-level features (such as the shape of specific objects) [5–7].

**Fig. 2:**
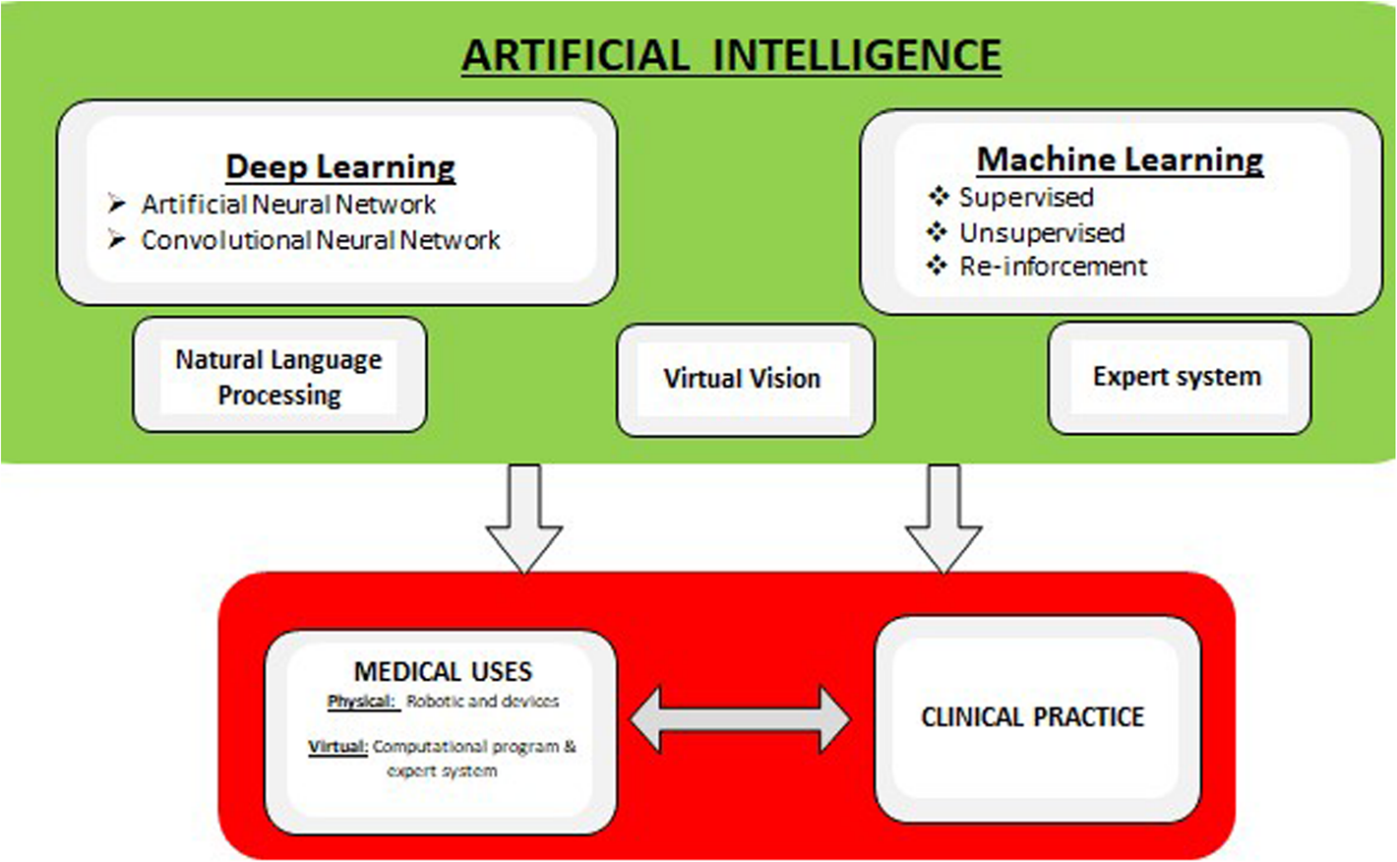
Components of artificial intelligence and use in the medical field

In recent years, with faster computer equipment and radiomics, AI has already made significant progress in medical imaging evaluation and radiological diagnosis of pulmonary diseases. Radiomics typically consists of five components: data selection, medical imaging, feature extraction, exploratory analysis, and modelling. Radiomics consist of extracting and analysing imaging features such as shape, texture or intensity of voxels from radiographic medical images using data-characterisation algorithms and relies on statistics. Radiomics is currently very much in use by pulmonary radiologists [8]. Figure 2 is a schematic representation of the various AI domains and interplay for medical usages.

### Current utilities

Initially, the AI performance and involvement was limited by low sensitivity, incomplete medical knowledge datasets and insufficient ability to integrate probabilistic reasoning. The chronological evolution history of AI and usage in the medical field is congregated in Table 1 [9]. The advances that have been already executed with the help of AI and DL are now well-designed in various aspects of pulmonary medicine but with limited usefulness in thoracic surgery. The significant medical field advancements that have been developed with the help of AI technique may be broadly categorised under these headings for thoracic surgical specialists:

1. Thoracic imaging interpretation and diagnosis making.
2. Histopathology or cytology assessment to aid in proper diagnosis.
3. Physiological measurements and bio-signal testing.
4. Virtual reality platforms and preoperative AI-based planning for lung resections. AI-based medical brain servers to interpret patient data and diagnosis making.
5. Robotic / Machine assisted surgery, including automatic camera handling devices, Robotic surgery platforms, and voice-controlled robotics.
6. Intraoperative usage of AI-based endoscopic systems for identification of tumours during surgery and virtual endoscopy.
7. AI-based algorithms for overall risk assessment and risk scoring with prognostication.
8. Surgical education and training.

**Table 1:** Timeline of major development in AI field with medical applications

Year	Development
**1950**	Alan Turing develops the “ Turing test”
**1956**	1st international conference on AI called “Dartmouth Summer Research Project on AI” conducted. John McCarthy coins the term “AI”
**1958**	John McCarthy creates the first full logical AI programme and described in technical paper “Programs with common sense”.
**1959**	The term “Machine learning” coined by Arthur Samuel.
**1961**	“Unimate” the first industrial robot, which worked on a General Motors assembly line in Ewing Township, New Jersey, USA.
**1964**	First chatbot “ELIZA” (natural language processing computer programme) developed by Joseph Weizenbaum.
**1966**	The first animated robot to reason about its actions, “Shakey -first electronic person” produced at Stanford University.
**1971**	A pilot research resource on computers in Biomedicine was funded at Rutgers University under Saul Amarel.
**1972**	“MYCIN” a clinical decision support consultation system, focused on appropriate management of patients who had infections, developed at Stanford University by Edward Shortliffe.
**1973**	“SUMEX-AIM” project (Stanford University Medical Experimental computer for Artificial Intelligence in Medicine) a national computer resource funded by the National Institutes of Health, USA started.
**1975**	The first National Institutes of Health, USA sponsored workshop conducted at Rutgers Research Resource on Computers in Biomedicine as the first in a series of workshops on AI in Medicine.
**1976**	AI based, Causal-associational network (CASNET) model characterises the pathophysiological mechanisms and clinical course of treated and untreated diseases for glaucoma.
**1978**	Herbert Simon earned a Nobel Prize for his “Limited Rationality Theory” which is an important work on AI.
**1980**	CADUCEUS has been developed as the knowledge-intensive expert system for medical diagnosis.
**1986**	First driverless Robot car, built in Munich under the direction of Ernst Dickmanns, drives up to 55 mph on empty streets. Barbara Grosz created the first computation model “Discourse” for the field of research.
**1997**	IBM’s Deep Blue Defeats Garry Kasparov in Chess.
**2000**	MIT’s Cynthia Breazeal develops “KisMet”, a robot that could recognise and simulate emotions.
**2007**	IBM begin development of Deep QA technology-based “IBM Watson” programme.
**2010**	Microsoft launched Kinect for Xbox 360, the first gaming device to track human body movement, using 3D camera and infra-red detection, creating virtual reality and enabling users to play their Xbox 360 wirelessly.
**2012**	Apple introduces Siri “Intelligent personal assistant”
**2014**	Amazon virtual assistance “Alexa “released.
**2015**	“Pharmabot” A Pediatric Generic Medicine Consulting chatbot developed.
**2017**	“Arterys”, cloud-based medical imaging software for cardiac MRI analysis get USFDA approval.
**2018**	First Fatal accident involving a Robotic car with a Pedestrian occurred.
**2020**	Microsoft introduced its Turing Natural Language Generation (T-NLG), which is the “largest language model ever published at 17 billion parameters.”

AI has been initially used in the diagnosis of lung nodules by use of Radiomics. Computer-aided diagnostics algorithms were developed initially as an assistance tool for radiologists to detect chest radiograph lesions. These algorithms were subsequently used for similar purposes for computed tomography (CT), magnetic resonance imaging (MRI) image interpretations, and the nuclear medicine field. These AI-based algorithms provide not only second opinions to assist radiologists but also save precious time. While the heavy workload of several chest x-rays or Chest CT films examination by radiologists for detecting precisely lung nodules or masses can be challenging tasks, AI-based algorithms and software can serve as valuable diagnostic aids, especially for suspicious lung nodules. By automating these simple tasks, radiologists are freed, and it demonstrates an improvement in human capital use by clinicians and synergistic cooperation with AI technology. In 2011, National Lung Screening Trial (NLST) for early detection of lung cancer by low-dose CT scan and in 2018, Netherlands Leuvens Longkanker Screening ONderzoek (NELSON) trials had provided an extensive data set to explore using AI. The NLST found a relative reduction in lung cancer mortality with low-dose CT screening of 20% with a 95% confidence interval (CI). The Nelson study is another randomised lung cancer screening trial using low dose CT that showed very promising results and is more favourable than the NLST results but suggests gender differences. Ardila et al. developed an alternative deep convolutional neural network for a complete assessment of low-dose CT volume focusing on concern regions of concern compared to prior imaging when available and standardisation against biopsy-confirmed outcomes [10]. Haitao Niu recently described the very promising experimental results of a visual feature extraction model based on AI with thoracic echo spectrum, and they established the effectiveness of this method compared with the latest approaches for radiological diagnosis of lung cancers [11].

We are also witnessing the recent development of various AI-based applications of digital pathology assessment for histopathological diagnosis. By researching the large archival databases of digital pathology images and detailed image analysis facilitate accurate diagnosis. It can also provide support from remote institutes for an expert second opinion [12].

Topalovic et al. have already demonstrated that AI-based algorithms might outperform the pulmonologists to interpret pulmonary function tests. AI-based auscultatory lung sound analysis of normal and adventitious lung sounds has been tried, but as these sounds are highly variable, henceforth, it is a challenge in generalising these algorithms based on decisions [13].

Most recently, Sadeghi et al. have presented the first pilot study about the development of an immersive virtual reality-based platform (PulmoVR) that enables personalised surgical planning for pulmonary segmentectomies. This is the first dedicated virtual reality-based segmentectomy planning tool that provides accurate surgical planning. But, this study contains only ten patients operated by two thoracic surgeons, so future clinical studies can further evaluate and demonstrate the clinical benefits of such technology. This is the classical procedure of using virtual components of AI practice in the preoperative planning field [14]. Previously, Yang et al. also described image-based software use for locating the nodules with the 3D images and coloured segments merger. The clearly labelled lung structure was utilised in a preoperative virtual segmentectomy and the subsequently planned thoracoscopic surgery [15].

In contradiction of the medical field, the thoracic surgical field has near negligible use of AI-based technologies, especially for benign disease management. As described above, in thoracic oncology patient management, the virtual component of AI is being used. In pure terms, surgeons are using the mechatronic or robotic branch of AI in a very limited manner and the term ‘surgical robots’ are often mistakenly interpreted in the AI context [16]. Although the term ‘robotic surgery’ is often applied to the da Vinci surgical system device-assisted surgery, it is a misconception of fully autonomous surgery since it cannot be self-programmed, nor can it make independent decisions to perform any surgical procedure, and characteristically, it is only a master-slave kind of assist device. In the exact terminology, current robotic devices are only assistance device with an autonomy of level-2 (on a scale of level 1 to 5 in the robotic science field). Other AI physical component-based, assistance devices are in use such as robotised endoscope positioner for a stable view of the operating field with the elimination of camera shake by a human assistant. It makes thoracoscopic procedures more ergonomic with less physical discomfort for the surgeon by saving movements and enhanced precision [17]. Another experimental development in the robotic surgical field started in 2005 is voice-controlled and lightweight; low cost parallel robot PARAMIS (PARAllel Robot for Minimally Invasive Surgery (MIS) in Centre for Industrial Robots Simulation and Testing (CESTER) within the Technical University of Cluj-Napoca, Romania by Prof. Doina Pisla and team. These are initial steps toward a multi-arm, low-cost robot with other additional modalities, which can be used in experimental laparoscopic surgery. The first porcine cholecystectomy has been successfully performed and reported by this team. The experimental results recommend PARAMIS as a potentially valuable tool for MIS. The cost of such a robot is only about 20,000 Euro, which is very cheap compared to the other commercial laparoscope holder devices or available assisting robots. But, this is still an experimental prototype model without any human use approval [18, 19]. In 2016, Shademan et al. used smart tissue autonomous robot (STAR) and compared various metrics of intestinal anastomosis in pigs (consistency of the suturing, the pressure at which anastomosis leaked, the number of mistakes or removing the needle from tissue, completion time and lumen reduction in intestinal anastomoses) between a supervised autonomous system STAR, manual laparoscopic surgery, and clinically used robotic-assisted surgery in pig experiments. The supervised autonomy with STAR surpassed robotic-assisted surgery, laparoscopic approach, and manual surgery based on some metrics. The interactive decision-making and execution between the surgeon and the STAR system made way for a new exciting and innovative possibility to augment surgeons. This study demonstrated that in future, this kind of autonomous human surgery is possible. The STAR system established the feasibility of performing pig intestinal suturing. Still, there has been no US Food and Drug Administration (USFDA) approved clinical, surgical application in humans despite further research so far [20, 21]. The development history of approved robotic surgery is summarised in Table 2 [22]. But with the rapid advent of AI in the future, it seems promising that the AI-based robotic system could carry out the operative intervention by itself with full autonomy of level-5 and self-adaptation to different operative scenarios like the upcoming AI-based Tesla fully self-driving autopilot car [23]. Considering this future possibility, the European Union have discussed the possible future circumstances in which a robot can take autonomous decisions. It was debated that by the traditional rules, it would not make it possible to ascertain the party responsible for providing compensation in case of an accident and require the party to make accountable for any damage it has caused. The solution suggested was to consider a definite legal status for robots as ‘electronic persons’ for making them guilty for any possible damage they may cause [24].

**Table 2:** Robotic surgery development history

Year	Development
**1921**	Czech playwright Karel Capek introduced the notion and coined the term “Robot” in his play Rossom’s Universal Robots
**1985**	First Robotic assist surgery performed by “PUMA 560” to obtain brain biopsy by Kwoh et al.
**1988**	Transurethral prostate resection performed by “PROBOT” at Imperial College London.
**1992**	Integrated Surgical Supplies Ltd. of Sacramento, CA, developed ROBODOC to machine the femur with greater precision in hip replacement surgeries—first surgical robot approved by the USFDA.
**1993**	AESOP (made by Computer Motion company) telescope manipulator introduced.
**1995**	Intuitive surgical company is founded.
**1997**	First laparoscopy procedure is performed by a Robotic system.
**2000**	Da Vinci (Intuitive Surgical) and ZEUS (Computer Motion) surgical systems approved by FDA.
**2001**	First distant transatlantic robot-assisted procedure (cholecystectomy) done.
**2003**	Computer Motion and Intuitive Surgical merge into one company. ZEUS company is discontinued
**2004**	“Spine Assist” device by Mazor Robotics Ltd., a miniature robotic guidance system received USFDA approval for spinal procedures.
**2007**	“Sensei-X” device developed by Hansen Medical company received USFDA approval for intravascular procedures.
**2012**	23,000 hip and knee procedures conducted globally with “MAKO” robotic system by The Stryker company.
**2013**	As reported, Over 500,000 surgical procedures performed worldwide with the da Vinci system.
**2016**	In the USA, surgical figures came out that over half of prostatectomies and a third of hysterectomies are performed robotically.
**2019**	Over 5000 da Vinci surgical systems installed worldwide and approximately 6 million surgeries conducted globally.

Recently, AI-based technology development in the intraoperative surgical field in clinical trials is also shown for easy identification of tumours and margins with the help of AI-based diagnostic algorithms with in vivo optical imaging for quantitatively analysing images [25].

Santos Gracia et al. evaluated an AI-based artificial neural network’s performance for predicting cardio-pulmonary morbidity after Lung resection for non-small cell lung cancer (NSCLC). In this study, sensitivity and specificity for predictions were 0.67 (95% CI 0.49–0.79) and 1.00 (95% CI 0.97–1.00), respectively. Similarly, Esteva et al. used four different probabilistic Ann’s models, trained by data from 113 patients of NSCLC. As we all know, prognosis plays an essential role in patient management in thoracic oncology surgical practice because it implies the efficacy of the surgery with an overall impact on the patient’s short- and long-term outcomes. AI-based systems have also shown promising results and assistance tools to decision-making for thoracic surgery patients by evaluating their surgical risk and estimating their prognosis [26]. AI-based computer modelling systems are now an established powerful tool for patient-tailored treatment in the pulmonary medicine field.

Another potential use of AI is in surgical education and training by creating virtual reality platforms and different clinical scenarios. This technology can be used for surgical skill assessment and optimising the training of surgeons with an objective evaluation of the surgical trainees [27].

### Challenges

AI and DL usages in medical fields are still very challenging because of difficulties for conventional statistical analysis methods such as logistic regression to isolate and confirm relationships between predictors and outcomes, especially when relationships are nonlinear and with many individual and operators variables. The quality of the AI’s output or answers largely depends on the available data’s quality and amount. Moreover, most datasets include a large amount of information and various inputs, so the information provided by the data may not allow to get a single best output.

The applicability of various AI algorithms across other medical fields is critically dependent on multiple factors such as exact representativeness of included populations, missing data links, and outliers. For example, any specific thoracic disease that affects any population group in one continent may not be existent with the same epidemiologic characteristics in another continent or even subcontinent area population. Therefore, under-representation of a particular group of people may have a risk of selection bias which may impact final generalised predictions. By inputting low socioeconomic status as a significant risk factor for premature mortality, AI algorithms may also be biassed against patients of a particular ethnicity or socioeconomic status due to incomplete baseline data, which may widen the gap in health outcomes. Embedded prejudice can only be prevented by massive, robust datasets of a true representative cross-section of all populations. Another example relates to the inequalities of healthcare delivery and the hefty financial expenses of these services. Vital Surgical decision making involves the complex analysis of a patient’s clinical assessment with physician intuition based on clinical experience while considering all relevant medical history, adjustable risk factors, and possible complications with the patient’s emotional expectations. Each decision-making step introduces considerable variability and opportunities for probable errors, especially in critical thoracic surgical condition management.

Nevertheless, external validation by using an independent dataset is of utmost critical before implementation in real-world clinical practice with inherently opaque AI algorithm black-box models. In the current era of evidence-based medicine, clinical decisions are based on the data provided by large-scale randomised clinical trials. Another hurdle of the generous use of AI in healthcare is the lack of randomised and prospective validation studies with difficulty improving an algorithm's performance on a theoretical basis only [28].

One illustration of iatrogenic harm related to AI-based algorithms is already on records by hundreds of hospitals worldwide using the IBM Watson supercomputer system for recommending cancer treatments in 2018. Algorithms based decisions on a small number of artificial theoretical, fictional cases using limited input from oncologists resulted in wrong output, and treatment recommendations. This faulty finding required systemic debugging, exhaustive audits, extensive iteration and evidence from other robust, and prospective validation studies before algorithms are widely implemented in actual clinical practice.

Although their accuracy is impressive, a drawback of AI and DL is also their lack of interpretability. The features used to distinguish between data categories are not readily translated into verbal or visual rules that any human can easily understand. Inculcating AI into our data process methods and make medical decisions may also take time. Prospective validation studies, particularly in clinical settings, are still lacking, and there is some valid fear that over-reliance on AI-based technologies might result in de-skilling our human workforce. There may be unsatisfactory outcomes, especially if initial algorithms are poorly generalisable or built on unstructured or unreliable data. In addition, due to the lack of technical awareness and its potential usefulness, many healthcare professionals identify AI as a threat to future medical jobs [29].

As of now, recent developments are focused more on the virtual component of AI, considering the human brain as a replica model. In surgical fields, especially thoracic surgery, which also need keen visual perceptions, fine tactile sensation, and delicate hand dexterity with hand-eye coordination as key significant components for successful surgery completion, the physical component of AI is still lagging behind.

There is also word of cautions by Notable scientist Stephen Hawking, Microsoft founder Bill Gates, history professor Yuval Noah Harari, and Space-X founder Elon Musk. They have expressed serious concerns about the possibility that AI could evolve to the extreme uncontrollable point, with Stephen Hawking even speculating that this could “spell the end of the human race as once humans develop AI, it will take off on its own and redesign itself at an ever-increasing rate and humans, who are limited by slow biological evolution, couldn't compete and would be superseded”.

### Future ahead

AI-based various machineries are already an integral part of our daily lives. As AI encompasses all three modalities and increasing computational power with the massive datasets that make up ‘big data’, it is also encouraging for healthcare applications. As our ability to manipulate images, language and speech increase with the help of AI and with the advent of new hardware as neuromorphic chips and quantum computing, larger datasets will become easily analysable. Other medical specialities such as radiology and pathology are most receptive to AI-based applications. As of now, more than 75 AI-based algorithms are approved by the USFDA, and AI-based medical imaging investments in the radiology and pulmonary medicine field have grown exponentially to USD 1.17 billion in the last years. To our knowledge, after an extensive literature search, various algorithms related to pulmonary medicine have also received premarket approval from USFDA recently in the previous two years, and several more are CE-marked (mandatory regulatory marking for products within the European Union economic area). Lagging growth in the thoracic surgery field compared with some other medical fields also warrants a need for increased responsiveness among doctors of these specialities for this advancement [30].

Several commercial establishments and academic institutions are in the phase of building massive clinical datasets with the hope that DL derived algorithms will favourably impact disease prediction, prevention, diagnosis, treatment and boost in healthcare-related economics. The recent development of medical brain platforms by AI technology giants as Alibaba and google is also under process and creating the latest attempt for digital revolution of health care.

The future of AI-related medical applications also depends on safety, confidentiality and data security. There will be sceptical interest in using algorithms that may risk revealing patient identity in light of hacking, cyber thefts and various instances of data breaches even in the highest cybersecurity zones and legal privacy issues. There are also chances that clinician skill sets and judgments may be weakened from an overdependence on automated AI-based systems with possible increases in medical errors [31].

## Conclusion

The spectrum to which AI technology may change our attitudes to screening and diagnosing thoracic diseases and the overall healthcare scenario is quite massive and full of potential. It is necessary to improve knowledge in this new field and understand the various means by which AI technologies could impact thoracic surgical practice and enhance collaboration with pulmonologists, pathologists and radiologists. Currently, genuinely autonomous surgery by Robots does not exist, and no application seems likely shortly. Nonetheless, taking an interest in recent AI technology with its capabilities and progression in related medical specialities will allow us to improve patient management in various clinical scenarios and future research possibilities. Surgeons can also be strategic in close partnership with other medical specialists and AI engineers, AI designers, and hospital administration to assess AI’s relevance and corroborate its usage in their practice to augment better thoracic surgical care. In future, we should not embrace this as ‘human versus machine’ but rather a cordial partnership to further improve clinical outcomes. This knowledge enhancement is also a fundamental requirement as worldwide governments are increasing their funding in this promising field and changing their legislator conditions to promote AI's safe and secure introduction. The simplest and most recent example is the inclusion and approval of telemedicine use in various countries. With genuine concerns, clinical supervision of AI-based techniques and robotic procedures are an essential prerequisite for further development. However, there are likely to be several other advancements and technologies that may change this scenario in the near future.

## Data Availability

Not applicable

## Abbreviations

AI: Artificial intelligence
ML: Machine learning
DL: Deep learning
ANNs: Artificial neural networks
DNNs: Deep neural networks
CT: Computerised tomography
MRI: Magnetic resonance imaging
CI: Confidence Interval
MIS: Minimally Invasive Surgery
PARAMIS: Parallel Robot for Minimally Invasive Surgery
CESTER: Centre for Industrial Robots Simulation and Testing
STAR: Smart tissue autonomous robot
USFDA: United States Food and Drug Administration
USD: US Dollar
